# Thermosensitive hydrogel carrying extracellular vesicles from adipose-derived stem cells promotes peripheral nerve regeneration after microsurgical repair

**DOI:** 10.1063/5.0118862

**Published:** 2022-11-04

**Authors:** Shih-Heng Chen, Huang-Kai Kao, Jing-Ru Wun, Pang-Yun Chou, Zhi-Yu Chen, Shih-Hsien Chen, Sung-Tsang Hsieh, Hsu-Wei Fang, Feng-Huei Lin

**Affiliations:** 1 Institute of Biomedical Engineering, College of Medicine and College of Engineering, National Taiwan University, Taipei, Taiwan; 2 Department of Plastic and Reconstructive Surgery, Chang-Gung Memorial Hospital, Chang-Gung University and Medical College, Taoyuan, Taiwan; 3 Department of Chemical Engineering and Biotechnology, National Taipei University of Technology, Taipei, Taiwan; 4 Division of Biomedical Engineering and Nanomedicine Research, National Health Research Institutes, Miaoli, Taiwan; 5 Department of Anatomy and Cell Biology, National Taiwan University College of Medicine, Taipei, Taiwan; 6 Department of Neurology, National Taiwan University Hospital, Taipei, Taiwan

## Abstract

Peripheral nerve injuries are commonly occurring traumas of the extremities; functional recovery is hindered by slow nerve regeneration (<1 mm/day) following microsurgical repair and subsequent muscle atrophy. Functional recovery after peripheral nerve repair is highly dependent on local Schwann cell activity and axon regeneration speed. Herein, to promote nerve regeneration, paracrine signals of adipose-derived stem cells were applied in the form of extracellular vesicles (EVs) loaded in a thermosensitive hydrogel (PALDE) that could solidify rapidly and sustain high EV concentration around a repaired nerve during surgery. Cell experiments revealed that PALDE hydrogel markedly promotes Schwann-cell migration and proliferation and axon outgrowth. In a rat sciatic nerve repair model, the PALDE hydrogel increased repaired-nerve conduction efficacy; contraction force of leg muscles innervated by the repaired nerve also recovered. Electromicroscopic examination of downstream nerves indicated that fascicle diameter and myeline thickness in the PALDE group (1.91 ± 0.61 and 1.06 ± 0.40 *μ*m, respectively) were significantly higher than those in PALD and control groups. Thus, this EV-loaded thermosensitive hydrogel is a potential cell-free therapeutic modality to improve peripheral-nerve regeneration, offering sustained and focused EV release around the nerve-injury site to overcome rapid clearance and maintain EV bioactivity *in vivo*.

## I. INTRODUCTION

Peripheral nerve injuries often cause motor and sensory deficits, including atrophy of downstream muscles and loss of sensation or neuropathic pain of the innervated dermatome.^1^ Trauma to the extremities is the main cause of peripheral-nerve injuries and often attributable to traffic or industrial accidents and battlefield injuries.^1,2^ Consequently, peripheral nerve injuries are generally distributed among younger individuals, particularly those in their productive years.^1,2^ Functional recovery after microsurgical nerve repair is dependent on the distance between the trauma zone and target organ of the regenerated nerve.^3^ Despite considerable advancements in microsurgery, functional recovery after nerve repair can be unpredictable and incomplete.^4^ A clinical study regarding functional outcomes after repair of ulnar and median nerves revealed that only 51.6% of patients experienced return of muscle power to M4 or M5 grade, and only 42.6% reported satisfactory (S3+ to S4) sensory return.^5^ Therefore, adjunct treatments for peripheral-nerve regeneration after coaptation are essential to improve functional recovery.

Neurite regeneration after peripheral nerve repair is highly dependent on Schwann cells (SCs). Chromatolysis is programmed immediately after peripheral-nerve injury by degeneration of traumatized axons and their surrounding myelin. This process begins at the zone of trauma and progresses upstream to the closest node of Ranvier before extending downstream to the entire nerve in a process known as Wallerian degeneration.^3^ During this process, SCs phagocytose axonal and myelin debris to clean out endoneurial tubes^3^ and recruit macrophages to synergistically promote SC activity.^3^ Subsequently, emptied endoneurial tubes are refilled by aligned SCs to form bands of Büngner,^6^ providing channels to guide the outgrowth of neurites to their target organs.^6^ As SCs are key players in peripheral-nerve regeneration after injury, they have been proposed as an option for cell therapy during nerve coaptations.^7,8^ However, the immunogenicity of allogenic SCs limits their use, and autologous SC transplantation is hindered by prolonged expansion *in vitro* and morbidities resulting from the sacrifice of donor nerves, which limits the source for harvest in clinical scenario. Other promising routes to utilize bioactive cues of SCs include transplantation of SC-like cells,^9–11^ SC-derived biomaterials,^9,12^ or augmentation of local SC recruitment.^13–15^ SC-like cells require induction processes for stem cells of various origins to differentiate into SC-like cells and face the challenges to maintain their phenotype *in vivo.*^11,16^ SC-derived materials utilize the secreted factors or extracellular matrix of SCs, but may still be limited by the clinical availability of SCs, either allogenic or autologous. In addition, SCs exhibit different phenotypes (e.g., SCs of repair or mature phenotypes), and the secreted factors may work oppositely regarding axon regeneration and myelination,^17,18^ which require further study for elucidation. Therefore, the strategy of the present study is not focused on using exogenous SCs or related factors, but dedicated to recruit local endogenous SCs and promote their proliferation and migration in the trauma zone after microsurgical coaptation to improving nerve regeneration.

Adipose-derived stem cells (ADSCs) have been proposed to enhance peripheral nerve regeneration^19,20^ by promoting local cell activity via paracrine effects.^21–23^ Paracrine mechanisms can involve secreted factors,^24^ including extracellular vesicles (EVs).^25,26^ EVs are proteolipid bilayer spheroids under 200 nm in diameter that can transfer intercellular signals via proteins, nucleic acids, and lipids, influencing recipient cell function.^27,28^ ADSC-derived EVs can modify SC activity and promote neurite outgrowth via paracrine effects.^13,29^ Therefore, EVs may represent an alternative to cell therapy, enhancing local SC activity to promote neurite outgrowth. Conventionally, EVs are administered through systemic injection, which is challenged by rapid clearance of EVs from blood with compromised therapeutic effect;^30,31^ however, locally applied EVs also encounter rapid clearance and dilution by body fluid, resulting in a short halflife.^32,33^ On the other hand, peripheral nerve regeneration requires a relatively long healing time. Furthermore, various studies have proved that sustained delivery of EVs significantly improves outcomes *in vivo* compared to the bolus injection of EVs.^34,35^ Therefore, it is necessary to develop a carrier to offer steady and extended release of EVs to maintain bioactivity at the site of nerve injury and to further accelerate neurite outgrowth.

Hydrogels have been proposed as carriers with the potential to mimic the extracellular matrix and promote nerve regeneration.^36^ In addition, tuning the hydrogel to be thermosensitive allows simple intraoperative application and rapid solidification at room temperature, ensuring that it wraps around the repaired nerve for a more focused delivery of EVs. In the literature, hydrogels have been proposed to carry a comparatively lower amount of EVs to produce and sustain the intended effect for a certain timespan^37^ because hydrogels prevent the loaded EVs from being cleared by body fluid or local cells prematurely.^38^ Additionally, a more focused and concentrated delivery of EVs is allowed by placing the EV-loaded hydrogel directly at the target organ. Moreover, considering that surface molecules of EVs are negatively charged,^39,40^ the application of a positively charged thermosensitive hydrogel would increase its affinity for ADSC-EVs, thereby facilitating a controlled and steady release. Positive charge reportedly induces and promotes SC migration, which may improve nerve regeneration.^41^ Various materials provide an overall positive charge,^42,43^ including lysine, which has a high affinity for SCs^44^ and neurons.^45^ Furthermore, polysaccharides, including dextran, have been proven to be beneficial for peripheral-nerve regeneration by promoting neurite outgrowth.^46–48^

**FIG. 1.**
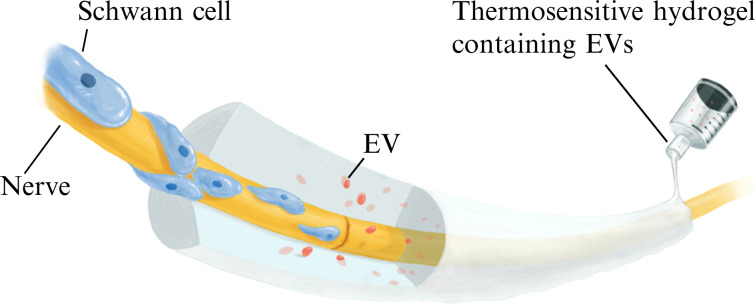
Study schematic. Extracellular vesicles (EVs) released from the PALDE hydrogel promote peripheral-nerve regeneration through the direct stimulation of neurite outgrowth and indirect promotion of Schwann cell migration and proliferation. Activated Schwann cells then form a synergistic effect with released EVs to facilitate nerve regeneration. The proposed PALDE hydrogel is designed for intraoperative use, improving functional outcomes after microsurgical nerve repair based on the above mechanism.

Thus, the present study describes the design of a thermosensitive hydrogel based on a pluronic-alginate mix polymer, as a cell-free therapeutic modality to promote peripheral-nerve repair (Fig. 1). In particular, the positively charged lysine–dextran component of the hydrogel will facilitate adsorption of ADSC-EVs. This hydrogel is expected to be thermo-responsive, solidify rapidly at body temperature, and steadily release EVs locally to promote nerve regeneration.

## II. RESULTS

### A. Characterization of ADSC-derived EVs

The isolated ADSC-EV particles generally exhibited circular morphology with a unimodal size distribution under scanning electron microscopy [SEM; Fig. S1(a)], and a central depression was observed via transmission electron microscopy [TEM; Fig. S1(b)]. The average diameter of ADSC-EVs was 119.3 ± 13.1 nm (ranging from 71.6 to 271.7 nm), with most particles measuring approximately 111 nm [Fig. S1(c)]. The concentration of the ADSC-EVs ranged from 10^8^ to 10^9^ particles/ml. Western blotting indicated the expression of EV-specific surface markers, including CD9 and CD63 [Fig. S1(d)].

### B. Characterization of thermosensitive hydrogels

#### 1. Rheology

**FIG. 2.**
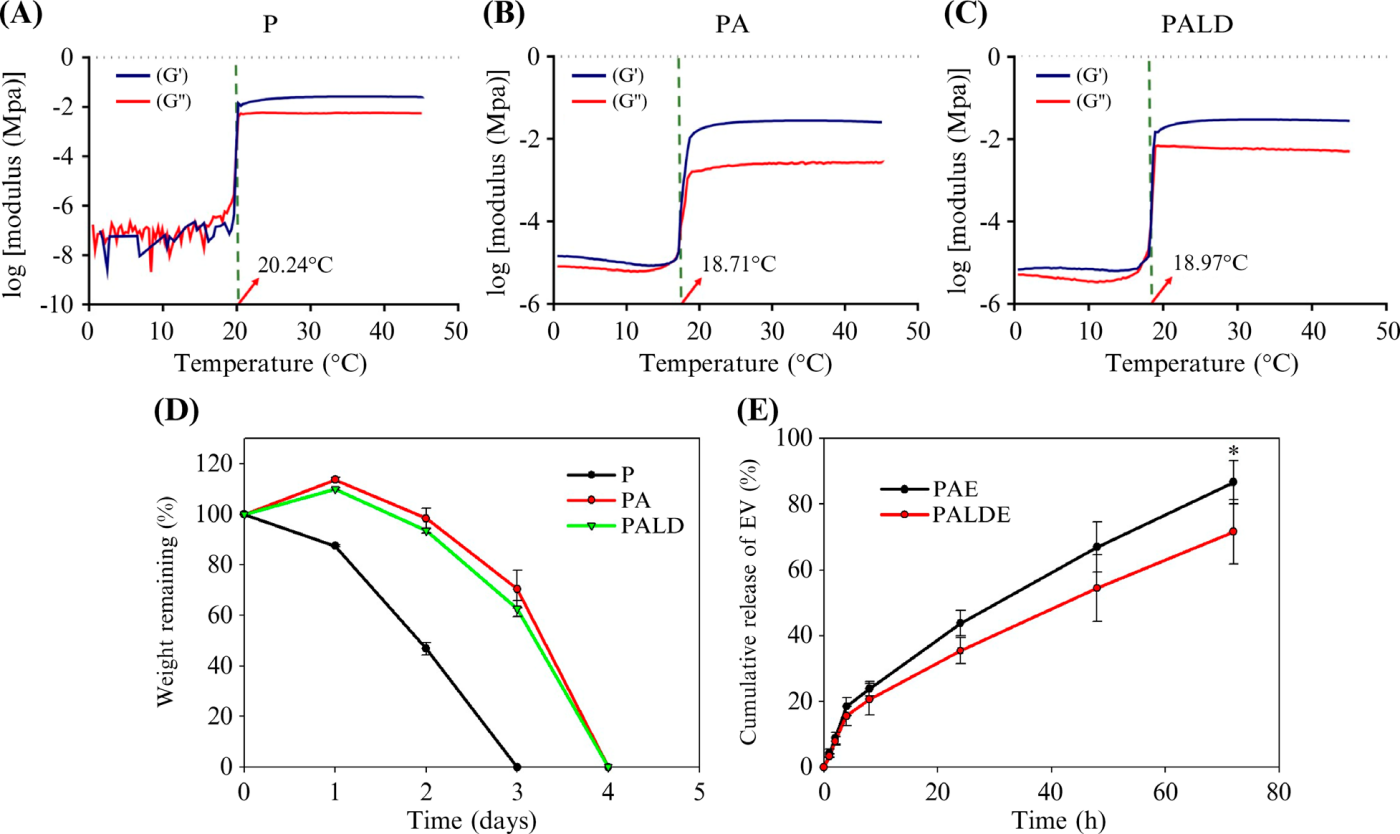
Rheological characterization, degradation, and release profile of various hydrogels. Rheological properties of (a) P, (b) PA, and (c) PALD hydrogels. (d) Degradation time of various hydrogels. (e) EV release profiles of PAE and PALDE hydrogels. Data are presented as mean ± standard deviation. ^*^p < 0.05. P, pluronic; PA, pluronic–alginate; PALD, pluronic–alginate–lysine–dextran; PAE, pluronic–alginate loaded with extracellular vesicles (EVs); PALDE, pluronic–alginate–lysine–dextran loaded with EVs.

The gelation temperatures of Pluronic (P), Pluronic-alginate (PA), and Pluronic–alginate–dextran (PALD) were 20.24, 18.97, and 18.97 °C, respectively [Figs. 2(a)–2(c) and Table S1], indicating that the addition of alginate and calcium chloride increased binding between molecules, which was reflected in the decreased gelation temperature. The gelation temperature of the PALD thermosensitive hydrogel was lower than the human body temperature, indicating an ability for rapid gelation during application in human tissues.

#### 2. Zeta potential

The zeta potentials of PA and PALD were −3.35 ± 1.81 and 6.83 ± 0.20 mV, respectively, implying that lysine-dextran yielded a positive charge (Table S1).

#### 3. Water content, degradation, and EV release

The water content (Table S1) of P, PA, and PALD hydrogels was over 75%, confirming their capacity to carry EVs. P hydrogels showed a degradation time of 3 days, whereas PA and PALD hydrogels lasted up to 4 days, reflecting increased molecular binding following the addition of alginate and calcium chloride [Fig. 2(d)]. The degradation of the hydrogels was performed with the gel immersed in phosphate buffer solution (PBS), and the process in the beginning included adsorption of water by the hydrogel, which resulted in swelling phenomenon and, thus, resulted in over 100% of the wet weight at the beginning of the test [Fig. 2(d)]. Pure Pluronic F127 was ruled out as an EV carrier based on the degradation test results.

The cumulative release of EVs was compared between PA and PALD loaded with EV (PAE and PALDE, respectively) gels to identify the effect of lysine-dextran on EV release [Fig. 2(e)]. From 0 to 8 h, both PAE and PALDE exhibited a surge in release (23.81% and 20.64%), likely related to the simple diffusion of EVs moving toward the surrounding PBS. However, from 12 to 72 h, the release rate decreased, and PALDE exhibited a more controlled release of EVs than PAE. This difference reached statistical significance at 72 h and thereafter (*p* < 0.05). The cumulative release of EVs from PAE and PALDE at 72 h was 86.68% and 71.64%, respectively. This result indicates that the positively charged lysine-dextran component of the hydrogel can facilitate EV adsorption for sustained EV release. Therefore, PALDE was chosen for further study.

### C. *In vitro* experiments

#### 1. Cytotoxicity of materials

Based on the ISO-10993 protocol, we used L929 cells to evaluate the cytotoxicity of the P, PA, and PALD hydrogels. The results are shown in Fig. S2; none of the tested thermosensitive hydrogels were toxic toward L929 cells.

#### 2. SC proliferation assay

**FIG. 3.**
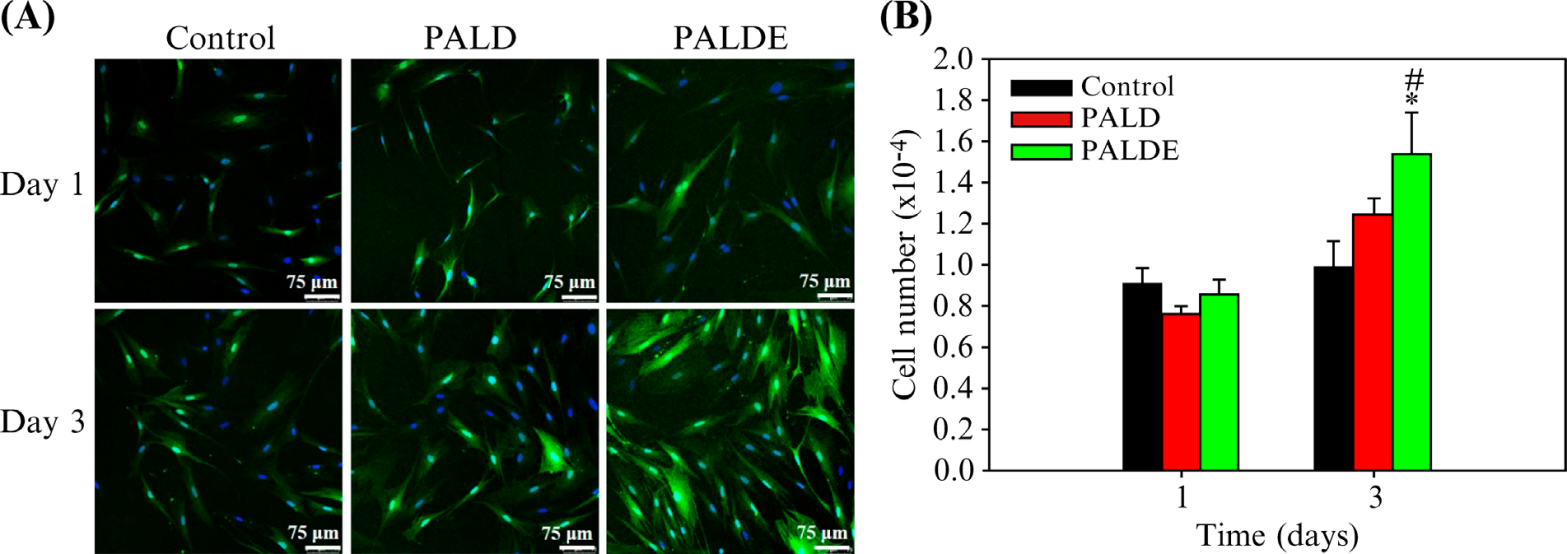
Schwann cell proliferation assay. (a) Confocal microscopic images of SCs stained with DAPI (4′,6-diamidino-2-phenylindole) (blue) and S100 (green) to observe SC proliferation on days 1 and 3. (b) Quantification of SC proliferation in control, PALD, and PALDE groups. Data are presented as mean ± standard deviation. ^*^p < 0.05 compared with the control group. ^#^*p* < 0.05 compared with the PALD group. PALD, pluronic–alginate–lysine–dextran; PALDE, pluronic–alginate–lysine–dextran loaded with extracellular vesicles.

No significant difference was observed between the PALD hydrogel and control in terms of cell proliferation. Proliferation in the PALDE group was significantly higher than that of the PALD hydrogel and in the control group (Fig. 3).

#### 3. SC migration assay

**FIG. 4.**
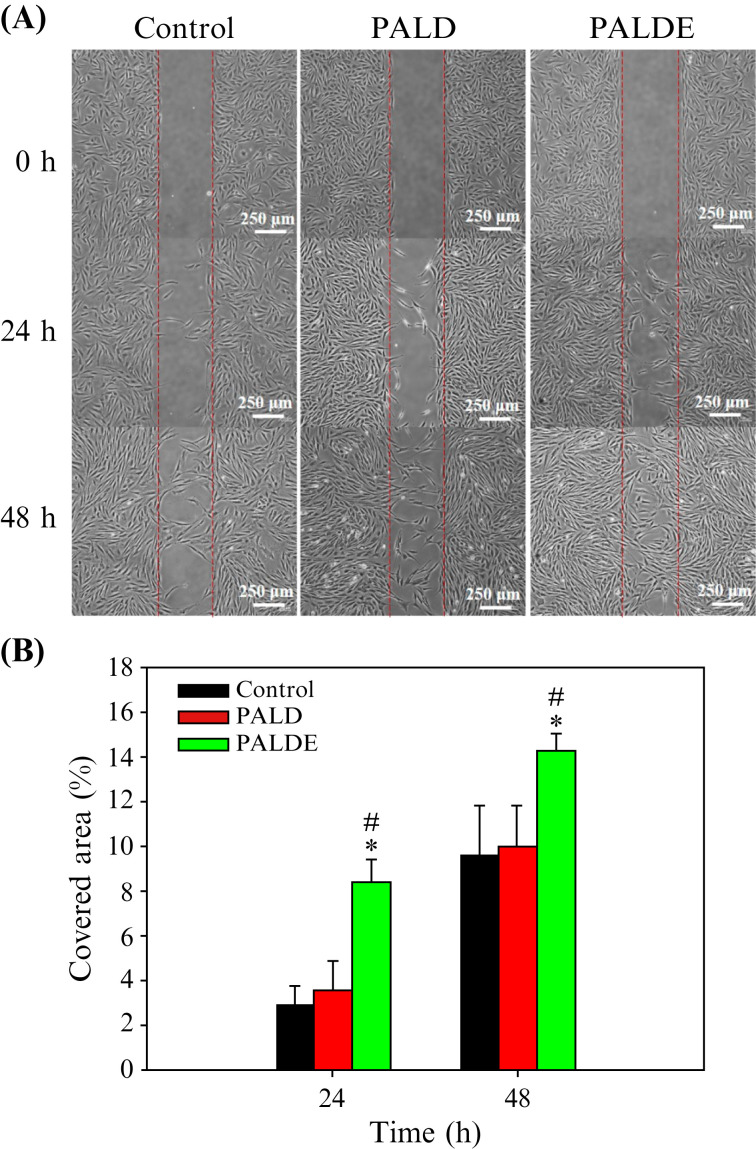
Schwann cell migration assay. (a) Migration of SCs among different groups using an optical microscope. The red lines represent the medial borders of the gap created in the assay. (b) Percentage of area covered by migrated SCs. Data are presented as mean ± standard deviation. ^*^p < 0.05 compared with the control group. ^#^*p* < 0.05 compared with the PALD group. PALD, pluronic–alginate–lysine–dextran; PALDE, pluronic–alginate–lysine–dextran loaded with extracellular vesicles.

The SCs in the PALDE group exhibited greater migratory ability than those in the other groups [Fig. 4(a)]. Quantification revealed that the PALDE hydrogel significantly promoted SC migration on days 1 and 2 compared with that in the PALD and control groups; the control and PALD groups did not exhibit significantly different migration levels [Fig. 4(b)].

#### 4. Neurite outgrowth assay

**FIG. 5.**
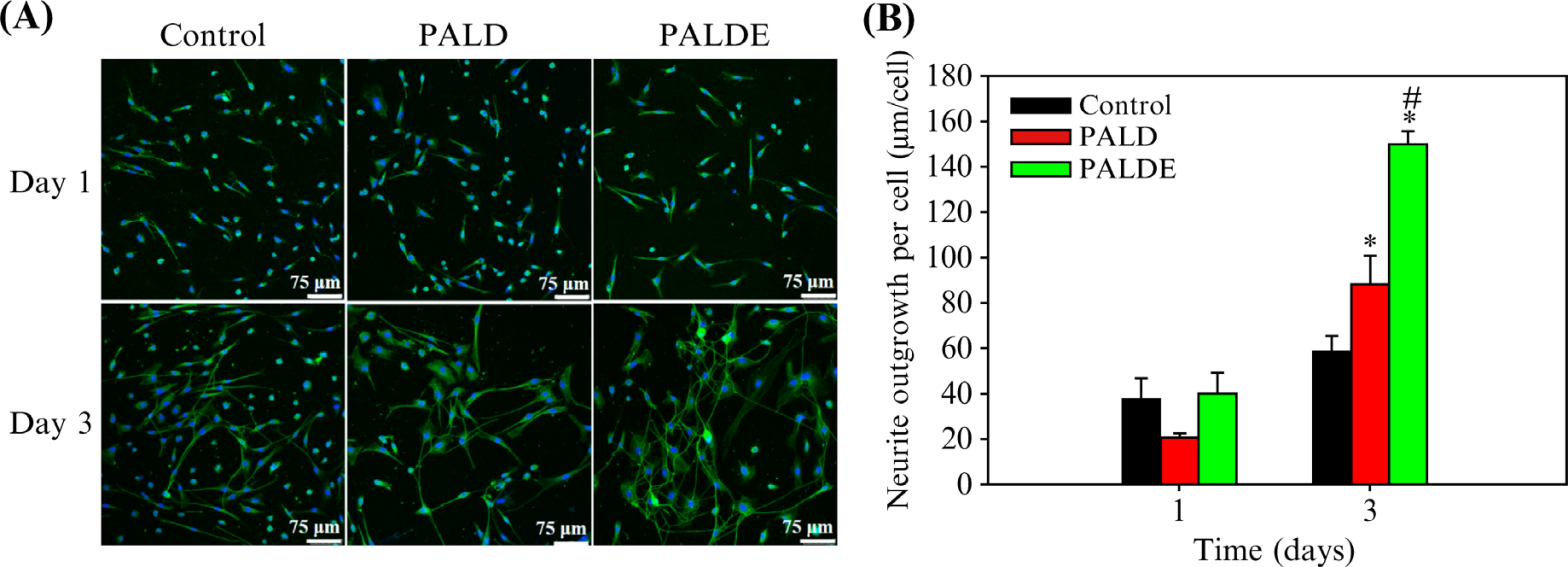
Neurite outgrowth assay. (a) The neurons were observed using antibodies against NeuN (blue) to reveal the nuclei and antibodies against β III-tubulin (green) to identify neurites. (b) Quantification of total neurite length in each group. Data are presented as mean ± standard deviation. ^*^p < 0.05 compared with the control group. ^#^*p* < 0.05 compared with the PALD group. PALD, pluronic–alginate–lysine–dextran; PALDE, pluronic–alginate–lysine–dextran loaded with extracellular vesicles.

Total neurite length in the PALDE group was significantly greater than that of the control and PALD neurites on day 3. PALD treatment resulted in greater neurite outgrowth than the control treatment (Fig. 5).

### D. *In vivo* experiments

#### 1. Functional outcome measurements

**FIG. 6.**
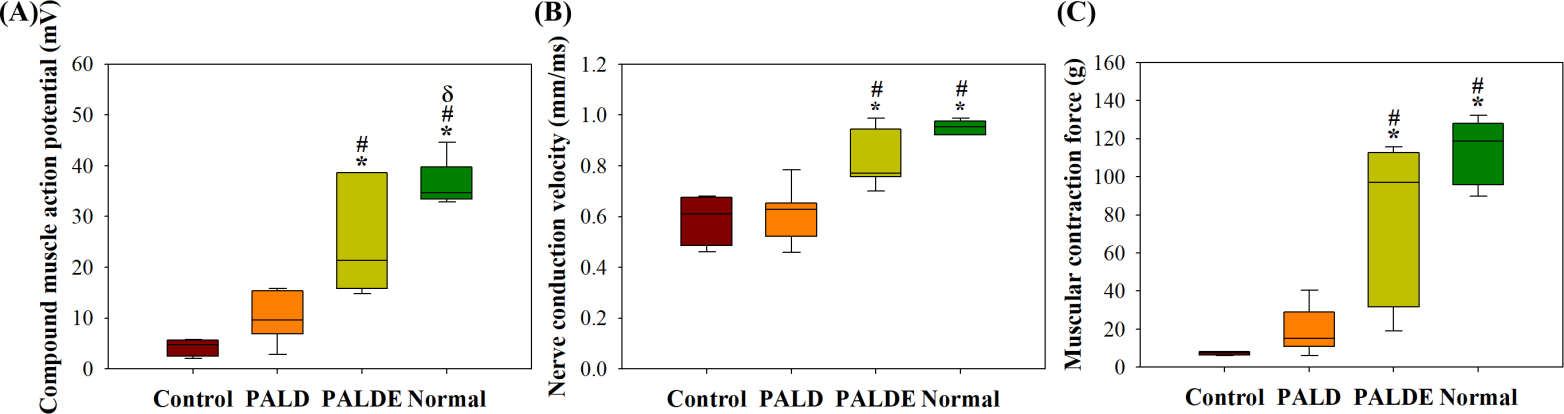
Functional recovery after sciatic nerve repair. (a) CMAP of the gastrocnemius muscle, (b) NCV, and (c) muscle contraction force. ^*^p < 0.05 compared with the control group; #*p* < 0.05 compared with the PALD group; δ*p* < 0.05 compared with the PALDE group. PALD, pluronic–alginate–lysine–dextran; PALDE, pluronic–alginate–lysine–dextran loaded with extracellular vesicles.

Following sciatic nerve repair, the compound muscle action potential (CMAP), nerve conduction velocity (NCV), and contraction force were significantly higher in the PALDE hydrogel group than in the control and PALD groups (Fig. 6). The PALD group exhibited higher CMAP and contraction force values than the control group; however, the difference was not significant.

#### 2. Percentage of muscle atrophy

**FIG. 7.**
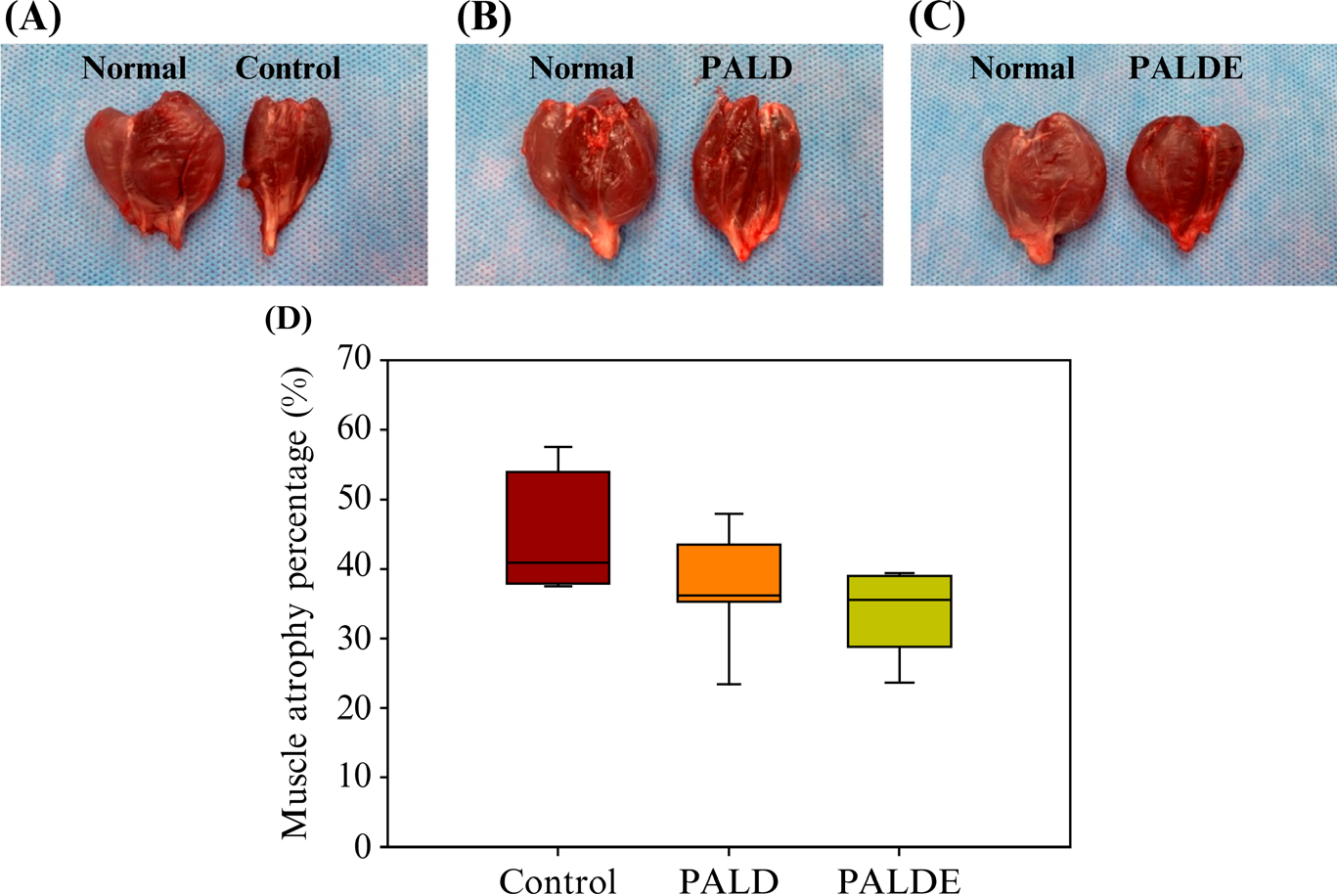
Comparison of muscle atrophy percentage among groups. Lower leg muscles from healthy and experimental sides in (a) control, (b) PALD, and (c) PALDE groups. (d) Quantification of muscle atrophy in different groups. PALD, pluronic–alginate–lysine–dextran; PALDE, pluronic–alginate–lysine–dextran loaded with extracellular vesicles.

Muscle atrophy in the PALDE-treated group was less severe than that in the control and PALD groups, although the difference was not significant (Fig. 7), suggesting that the EVs promoted nerve regeneration and subsequent skeletal muscle recovery.

#### 3. Fascicle diameter and myelin thickness of the downstream nerves

**FIG. 8.**
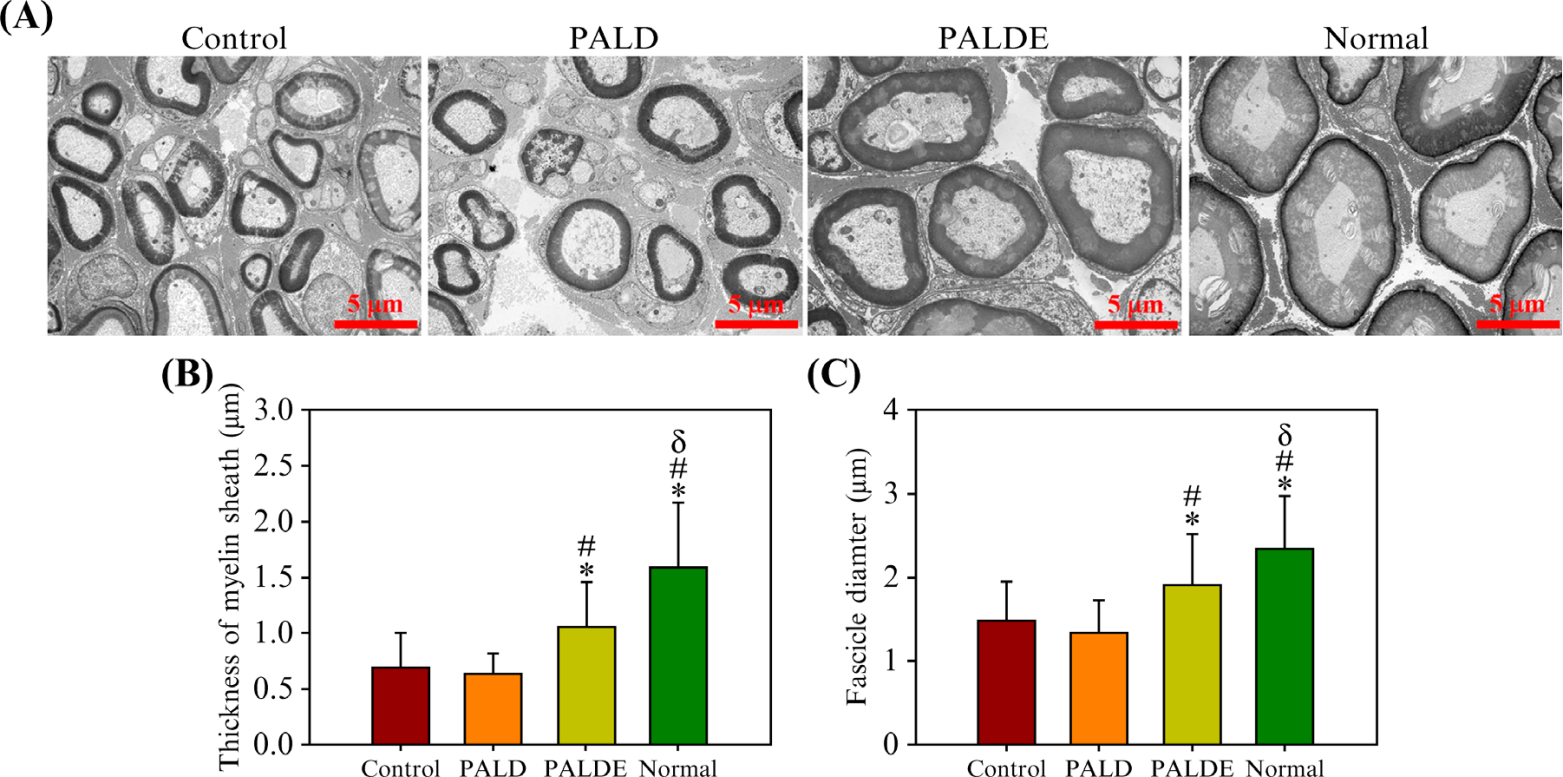
Morphology of regenerated nerves after sciatic nerve repair. (a) Downstream nerves in the control, PALD, PALDE, and normal groups, as assessed with transmission electron microscopy. Quantification and comparison of (b) myelin sheath thickness and (c) downstream fascicle diameter among various groups. ^*^p < 0.05 compared with the control group. ^#^*p* < 0.05 compared with the PALD group. δ*p* < 0.05 compared with the PALDE group. PALD, pluronic–alginate–lysine–dextran; PALDE, pluronic–alginate–lysine–dextran loaded with extracellular vesicles.

The quality of neural regeneration could be reflected by the fascicle diameter and myelin thickness of the downstream nerves, which is consistent with the results of electroneuromyography, because these two parameters are positively correlated with NCV.^49–51^ The PALDE group exhibited a downstream nerve fascicle diameter of 1.91 ± 0.61 μm and a myelin thickness of 1.06 ± 0.40 μm. In the PALD group, the fascicle diameter was 1.32 ± 0.42 μm and the myelin thickness was 0.63 ± 0.18 μm. In the control group, the fascicle diameter was 1.49 ± 0.48 μm and the myelin thickness was 0.69 ± 0.31 μm. In the healthy tissue, the fascicle diameter was 2.35 ± 0.64 μm and the myelin thickness was 1.59 ± 0.59 μm (Fig. 8). The values in the PALDE group were significantly higher than those in the PALD and control groups.

## III. DISCUSSION

### A. Benefits of using EVs

Owing to various obstacles in clinical cell therapy, the development toward cell-free therapies is a promising goal for improving the outcomes of peripheral-nerve repair. In the present study, EV was used as a modality to transduce paracrine signals originated from ADSCs; the thermosensitive hydrogel prolongs this signal stimulation via controlled and steady release. EVs modify recipient cell behavior through various mechanisms, including the activation of cell surface receptors via protein or lipid ligands and the incorporation of EV membrane contents into the target cell membrane with subsequent delivery of bioactive molecules.^52^ ADSCs have been tested for peripheral-nerve regeneration in various studies;^23,53^ however, the effect of ADSC-derived EVs on nerve regeneration has not been clarified until recently.^29,54^ Chen *et al.* demonstrated that ADSC-EVs not only increase the activity of SCs but also directly promote neurite outgrowth,^29^ which supports our findings. Considering that systemically administered EVs are quickly cleared *in vivo*,^30,55^ the local delivery of EVs with biomaterials has become a logical and effective alternative to utilize the potency of ADSC-EVs to promote peripheral-nerve regeneration. Based on the results of the present study, the proposed thermosensitive hydrogel preserved the effects of ADSC-EVs in both *in vitro* and *in vivo* studies, which supports their potential clinical application and our hypothesis that released EVs exert their effects on neurites and SCs.

### B. Schwann cell proliferation

During the entire process of peripheral-nerve regeneration, the successful proliferation of SCs is crucial to guide Wallerian degeneration, secrete substrates for neurite outgrowth, form bands of Büngner to direct neurite sprouting toward the target organ, and construct a myelin sheath to facilitate conduction.^6,56,57^ Impaired SC proliferation can hinder axonal regeneration,^58,59^ whereas ADSC transplantation can promote peripheral-nerve regeneration.^14,19^ In fact, the regenerative effects of transplanted ADSCs are related to EVs.^13,60^ Haertinger showed that the beneficial effect of EVs on SC proliferation is dose- and time-dependent.^13^ In the present *in vitro* study, PALDE did not cause significant promotion on SC proliferation until day 3, which partially reflected Haertinger's findings, and partly stemmed from the slow release of EVs from PALDE (20.64% on day 1 and 71.64% on day 3).

Furthermore, previous studies have demonstrated that polysaccharides can promote SC proliferation, thereby improving peripheral-nerve regeneration.^46,61^ In addition, a positive surface charge can promote the attachment and proliferation of SCs.^62^ Hentz *et al.* modified the surface charge of a hyaluronan-based nerve conduit by coating it with positively charged polylysine, revealing increased SC proliferation compared with the conduit without charge modification.^44^ In the present study, the PALD hydrogel promoted the proliferation of SCs on day 3 compared with the control, which is consistent with the literature. The addition of EVs to the hydrogel, PALDE, significantly augmented the proliferation of SCs.

### C. Schwann cell migration

The migratory capacity of SCs is crucial during nerve injury and regeneration^46,63^ as Wallerian degeneration and subsequent formation of Büngner bands require SC downstream migration.^6,63^ Additionally, SCs must relocate proximally to reach, and interact with, healthy axon stumps and guide neurite outgrowth.^64,65^ The present study revealed that the EVs released from PALDE hydrogel significantly promoted SC migration *in vitro* compared with the hydrogel without EVs and the control, which supports the hypothesis that the released EVs positively affect SC migration, and this effect was well-maintained by the proposed hydrogel carrier. However, it remains unclear by which mechanism EVs exert their effect on SC migration. The sphingosine 1-phosphate (S1P) receptor has been reported to trigger the rearrangement of the cytoskeleton and stimulate the migration of rat SCs,^66^ and S1P-enriched EVs have been shown to promote cell migration.^67^ Moreover, fibroblast growth factor 5 upregulates N-cadherin expression to promote SC migration through FGF (fibroblast growth factor) receptors.^68^ Although these preliminary findings suggest that EVs exert their effect on SC migration via certain receptors,^69,70^ further investigation is necessary to clarify the underlying mechanism.

### D. Neurite outgrowth

The effect of ADSC-EVs on neurite outgrowth has not been elucidated; however, Bucan *et al.* showed promising results in their study, though not reaching statistical significance.^54^ Our *in vitro* study showed significant promotion of neurite outgrowth by PALDE on day 3. In addition, our *in vivo* study showed significant improvement in functional outcomes as well as improvement in fascicle diameter and myelin thickness in the PALDE-treated group. In the study by Bucan *et al.*, they injected ADSC-EVs into the crushed sciatic nerve, whereas in the present study, the EVs were gradually released from the hydrogel. We propose that the more significant effect of our ADSC-EVs on neurite outgrowth and in the *in vivo* study is related to a prolonged release of the EVs from the hydrogel. However, it remains unclear whether the EVs have time- and dose-dependent effects on neurons.

Additionally, hydrogel degradability has been proposed to facilitate neurite outgrowth.^71,72^ Studies have revealed that the degradation process triggers the mechanotransduction of local cells and modifies cell behavior.^73,74^ Tang *et al.* demonstrated that cells in degradable hydrogels exhibit a more spreading morphology compare to those in non-degradable ones,^75^ which implied the modification in cytoskeletal tension and adhesion ligands and explained possible effects on neurites.^74^ Furthermore, the positive surface charge of the material and its effect on neurite outgrowth have been discussed,^76,77^ and the theory was that most cells present a negative charge^76^ and are prone to affinity with cationic materials; for example, chitosan has been described to facilitate attachment of neurons and increase axon outgrowth.^45^ In addition, certain polysaccharides containing mannose or glucose residues have been shown to improve axon sprouting.^78–80^ This study incorporated lysine-dextran to utilize both the positive charge of lysine and polysaccharide characteristics of glucose-based dextran. Although the detailed mechanism through which lysine-dextran exerts its effects on neurites is under investigation, our *in vitro* results suggest that PALD hydrogels promote axon regeneration directly and secondarily through effects on SCs, and that the addition of EVs in the PALD hydrogel further improves axon outgrowth.

### E. Functional outcomes

Anatomically, the motor neurons of the peripheral nerves lie in the spinal cord and send out nerve fibers to travel through the extremities to reach target muscles.^81^ Although PALDE promoted neurite outgrowth *in vitro*, its effectiveness in clinical scenarios is unclear as the released EVs can only exert effects on nerve fibers, rather than on neurons themselves, after intraoperative application. Therefore, *in vivo* functional outcomes are vital to peripheral nerve-related research. In animal experiments, when the nerve is stimulated, its motor units and muscle fibers generate an electric potential. The collective electric potential is also known as the CMAP. High CMAP values represent many muscles and motor endplates that can be recruited by the repaired nerve, indicating nerve regeneration. NCV is the velocity of the signal impulse spread along the nerve and is positively correlated with the quality and extent of myelination.^49,50^ The gastrocnemius and soleus muscles are innervated by the distal division of the sciatic nerve. Denervation and atrophy of these muscles begin owing to transection of the sciatic nerve and do not stop until reinnervation reaches the target muscles with endplate regeneration. Thus, the contraction force produced by these muscles is considered a measure of functional recovery after nerve repair. That is, an increased contraction force represents better motor return, which is also reflected by the results of muscle atrophy percentage.^82^ *In vivo* data from the present study, including functional and TEM evaluations of downstream nerves, are consistent. These data, together with the *in vitro* results, suggest that the intraoperative application of the PALDE hydrogel can improve the outcome of nerve repair, yielding superior electrical conduction to conventional nerve repair or the application of hydrogel without EVs. This is likely owing to the promotion of neurite outgrowth and SC activities by EVs, which could stem from bioactive cues;^13^ this deserves further study in the future.

## IV. CONCLUSIONS

This study demonstrated the therapeutic potential of cell-free therapy on peripheral-nerve regeneration by showing the effect of an EV-loaded thermosensitive hydrogel using *in vitro* and *in vivo* models. The PALDE hydrogel directly stimulated axon outgrowth and promoted SC proliferation and migration, suggesting a secondary effect on neurite outgrowth by influencing SC behavior. Moreover, the application of the PALDE hydrogel in a sciatic nerve repair model highlighted positive effects on CMAP, NCV, and muscle contraction force, along with a relative decrease in muscle atrophy. Downstream nerves in the PALDE group exhibited larger fascicle diameters and thicker myelin sheaths under TEM, consistent CMAP and NCV findings. Thus, the application of the PALDE thermosensitive hydrogel around repaired nerves provides a cell-free therapy with bioactive cues from ADSC-derived EVs to promote peripheral-nerve regeneration, representing a potentially practical design in clinical scenarios. However, the mechanism underlying the action of ADSC-EVs on neurons and SCs was not demonstrated herein. Furthermore, the underlying pathways and bioactive cues within EVs require further elucidation in future research.

## V. METHODS

### A. Preparation of EVs derived from ADSCs

To collect ADSC-derived EVs, ADSC culture medium was replaced with exosome-depleted Dulbecco's modified Eagle's medium (DMEM) supplemented with 10% exosome-depleted fetal bovine serum (FBS), 2 mM l-glutamine, and 100 U penicillin/100 U streptomycin when ADSCs reached 75%–85% confluence (passage 6–8). Supernatants were collected after 72 h of culture. EVs were isolated using size-exclusion chromatography with qEV columns (Izon, Cambridge, MA, USA) at 4 °C, following the manufacturer's protocol. Nanoparticle tracking analysis, western blot, electron microscopy, and Bradford assays were used for characterization and quantification of collected EVs (see the supplementary material).

### B. Thermosensitive hydrogel preparation and examination

Pluronic F127 (P) was used as the base of the hydrogel, and alginate with or without lysine–dextran in various combinations was added to form pluronic–alginate (PA) and pluronic–alginate–lysine–dextran (PALD) hydrogels (Table S2). The hydrogels were dissolved in ddH_2_O at 4 °C, and calcium chloride was added. The mixtures were dispersed by gentle stirring in de-ionized water. The resulting hydrogels were stored at 4 °C for subsequent experiments. Based on the results regarding effective dose of EV (Fig. S3), the EVs (3.6 mg/ml) were mixed in a ratio of 1:2 with PA or PALD hydrogels with stirring at 4 °C for 1 h, resulting in PAE and PALDE hydrogels.

### C. Characterization of thermosensitive hydrogels

#### 1. Rheometry

The change in viscosity of P, PA, and PALD thermosensitive hydrogels with respect to temperature was quantified using an HR-2 rheometer (TA Instruments, New Castle, DE, USA) and a Peltier plate temperature system. A 40-mm steel cone geometry with a 2° angle was used for evaluation. The rate of temperature increment was 2 °C/min, with a 30 s break before each measurement, and the stress was maintained steadily at 0.1 Pa at each temperature, with angular frequency set at 1 rad/s. The storage and loss moduli were obtained during each measurement, and the ratio of the loss modulus to the angular frequency was calculated to determine the viscosity of the hydrogel.

#### 2. Zeta potential

PA and PALD hydrogels were first diluted to 1% w/v of overall polymer concentration with de-ionized water. The pH was titrated to 7.4, and the temperature was set to 25 °C before measuring the zeta potential using a 90Plus/BI-MAS instrument (Brookhaven Instruments Corp., Holtsville, NY, USA).

#### 3. Degradation

Degradation tests were performed in PBS (pH 7.4) solution. The initial wet weight (Wi) of 1 ml of hydrogel was measured, followed by immersion in 2 ml of PBS at 37 °C for 1 week. At predetermined times, the PBS was removed, and the wet weight of the hydrogel (Wt) was obtained. The percentage degradation was calculated using the following equation:

$$\mathrm{Degradation}\;\left(\%\right)=\left[\frac{\mathrm{Wi}-\mathrm{Wt}}{\mathrm{Wi}}\right]\times 100.$$ (1)

#### 4. Water content

The water content of the equilibrated hydrogels was calculated based on Eq. (2), where W_swollen_ and W_dry_ represent the mass of a hydrogel in its swollen and dried states, respectively,

$$\mathrm{Water}\;\mathrm{content}\;\left(\%\right)=\left[\frac{W_{\mathrm{swollen}}-W_{\mathrm{dry}}}{W_{\mathrm{swollen}}}\right]\times 100.$$ (2)

#### 5. Release profile of EVs

At 37 °C, 1 ml each of solidified PAE and PALDE was immersed in 2 ml of PBS, and the solution was sampled at predetermined time points (8, 12, 24, 48, 72, and 96 h) to quantify the protein content. Bradford assays were conducted according to the manufacturer's instructions to observe the release of EVs from the hydrogels.

### D. Animals

All animal experiments and procedures were performed in accordance with the Guide for the Care and Use of Laboratory Animals issued by the Animal Research Committee of the Chang-Gung Memorial Hospital (IACUC, No. 2021062401), which is in accordance with the Guidelines for the Care and Use of Laboratory Animals published by the US National Institutes of Health (NIH Publication, eighth edition, 2011). Female adult Sprague Dawley rats were used and given *ad libitum* access to water and food under a 12 h light/dark cycle.

### E. *In vitro* cell experiments

#### 1. Cell cultures

The sciatic nerves of female Sprague Dawley rats (6 weeks old) were harvested for the isolation of primary SCs. Cell culture was performed using a specific medium following the protocol described by Kaewkhaw *et al.*^83^ SCs were maintained in flasks coated with 1.5 *μ*g/cm^2^ poly-L-lysine and filled with DMEM supplemented with 10% FBS and 1% penicillin/streptomycin. SCs between passages four and seven were used for *in vitro* experiments.

Primary neurons were isolated from the dorsal root ganglion (DRG) of neonatal rats based on the protocol described by Burkey.^84^ The DRG neurons were cultured in Neurobasal-A medium (Gibco) supplemented with 2% B27, nerve growth factor (50 ng/ml), and streptomycin and penicillin (100 U/ml) at 37 °C with 5% CO_2_.

#### 2. Cytotoxicity

Material cytotoxicity was tested based on the ISO-10993 protocol (see the supplementary material).

#### 3. SC proliferation test

SCs were seeded in 96‐well plates (3000 cells/well) coated with 0.01% poly-L-lysine. The wells were then filled with 10% FBS DMEM plus forskolin (5 *μ*M), N_2_ supplement (1% v/v), and bovine pituitary extract (20 *μ*g/ml). PALD or PALDE hydrogel (200 *μ*l) was placed in each well of a 24-well plate. The hydrogel was extracted with SC culture medium at 37 °C for 24 h to obtain the extraction medium. SC proliferation assays were performed in the control and experimental groups, including the PALD and PALDE groups, with 200 *μ*l of extraction medium added to each well. After culturing for 24 and 72 h, SCs were fixed with 4% paraformaldehyde and then stained with antibodies against S100. Immunohistochemical evaluation was performed using a confocal microscope. SC proliferation was quantified using the WST-1 assay kit after culturing for 24 and 72 h. The optical density at 450 nm was measured using an ELISA (enzyme-linked immunosorbent assay) reader, and cell numbers were determined using a calibration curve.

#### 4. SC migration assay

A gap-closure assay was performed to evaluate the efficacy of SC migration among groups using two-well silicone culture inserts (Ibidi GmbH, Martinsried, Germany) set with transparent dishes (Ibidi GmbH). The two-well inserts were first filled with 10% FBS DMEM and then seeded with SCs (1 × 10^4^ cells/cm^2^) for 24 h to form a confluent monolayer, which was visible through the transparent dish. After the removal of the inserts, a 500 *μ*m cell-free interval with clear demarcation was generated in the center of the dish. SCs were irrigated with PBS and then incubated in a serum-free medium at 37 °C. After replacing the serum-free medium with extracts of the control, PALD, and PALDE groups (in the same ratio as with the SC proliferation method), the migration of SCs from the medial edges of the interval across the 500 *μ*m distance was observed for 48 h. Images were taken using light microscopy and digitalized (Leica QWin, Germany) after 0, 24, and 48 h of migration; data were quantified using ImageJ.

#### 5. Neurite outgrowth assay

DRG neurons were seeded in 24-well plates (3000 cells/1.8 cm^2^) for 6 h until achieving quiescence. The culture medium was then replaced with extracts of the control, PALD, and PALDE groups (the medium/hydrogel ratio was 1 ml/200 *μ*l), and neurons were cultured for 24 and 72 h. The samples were fixed with 4% paraformaldehyde and stained with antibodies against β3‐tubulin and NeuN for the visualization of neurites and cell bodies of neurons, respectively, under a confocal microscope (Leica TCS SP8X STED, Leica, Hessen, Germany). Each plate was subdivided into four quadrants, and two visual fields in each quadrant were selected for the evaluation of neuron and neurite definition and overlap. Images of the selected visual fields were captured and digitalized (Leica Imaris 3D/4D Image Visualization & Analysis software, Leica). Total neurite length was quantified using Metamorph (Molecular Devices, San Jose, CA, USA).

### F. *In vivo* experiments

#### 1. Sciatic nerve repair model

The sciatic nerve of an 8-week-old Sprague Dawley rat was exposed through a longitudinal lateral thigh incision under general anesthesia using isoflurane (with the flow rate at 3% for induction, followed by 2% for maintenance). The sciatic nerve was transected at a point 1.5 cm proximal to its trifurcation. Direct coaptation of the transected nerve was performed with 9–0 nylon under a surgical microscope. In the surgical control group, the wound was sutured directly after sciatic nerve coaptation. In the PALD and PALDE groups, 0.5 ml of the respective hydrogel was applied around the nerve at the coaptation site before wound closure. For *in vivo* experiments, eight rats were used in each group.

#### 2. Functional outcome measurements

Three months after sciatic nerve coaptation, a second surgical procedure was performed on the rats for data collection. An IX-TA-220 recorder with integrated sensors and FT-302 force transducer (iWorx Systems Inc., Dover, NH, USA) were used to measure electroneuromyography and muscle contraction force of the repaired nerve and its motor units, following the protocols presented by Giusti^85^ and Nepomuceno.^86^ The procedure began with exploration of the sciatic nerve of the healthy limb, and the Achilles tendon was anchored to the force transducer using a 4–0 nylon suture to measure the isometric contraction force produced by the gastrocnemius and soleus. The gastrocnemius was fitted with a miniature bipolar receiver electrode for the simultaneous recording of the CMAP. The healthy sciatic nerve examined to evaluate supramaximal CMAP; the results represent the data of normal nerves. The amplitude that produced a supramaximal CMAP was recorded for subsequent stimulation of the experimental side (usually between 1 and 3 mA with a duration of 0.1 ms). Measurements were repeated on the experimental side, and the repaired sciatic nerve was exposed. Stimulation of the repaired nerve was performed at the point 1 cm proximal to the repair site using a bipolar stimulator. Once the nerve was stimulated, CMAP, nerve conduction latency, and maximal isometric muscle contraction force measurements were obtained. The distance between the nerve stimulator and CMAP receiver was measured and divided by the nerve conduction latency to generate NCV. After the experiment, the wounds were closed using nylon sutures. The animals were euthanized using carbon dioxide chambers with the CO_2_ flow rate at 70%.

#### 3. Muscle atrophy percentage

After euthanasia, the muscles of both healthy and experimental limbs were harvested from the tibia and fibula for weight measurements. The muscles harvested included the posterior compartment muscles (gastrocnemius, soleus, and tibialis posterior), anterior compartment muscles (tibialis anterior and extensor digitorum), and lateral compartment muscle (peroneus longus and brevis). The wet weight of the muscles from the experimental limb was divided by that of the healthy limb to generate the percentage of muscle atrophy.

#### 4. Histological evaluation of downstream nerve regeneration

The quality of downstream nerve regeneration was evaluated by axon diameter and myelin thickness using TEM (Hitachi HT7800, Hitachi, Tokyo, Japan). The tibial and peroneal nerves downstream of the sciatic nerve were harvested and fixed with 3% glutaraldehyde and 2% paraformaldehyde buffered with 0.1 M cacodylate (pH 7.4) at 4 °C and then post-fixed in 1% osmium tetroxide at pH 7.4. Next, a graded series of ethanol was applied to dehydrate the samples before embedding them in EPON-812 (Electron Microscopy Sciences, Hatfield, PA, USA). Sections measuring 80 nm in thickness were obtained and stained with uranyl acetate and lead citrate. Evaluation was aimed at the region 1–1.5 cm distally to the trifurcated sciatic nerve. Images were acquired with an electron microscope (Hitachi HT7800), and myelin thickness and fascicle diameter were measured using ImageJ.

### G. Statistical analysis

Data are presented as mean ± standard deviation. The means of the various groups were compared using one-way analysis of variance, and significant differences were defined by *p* < 0.05.

## SUPPLEMENTARY MATERIAL

See the supplementary material for materials and reagents, characterization of extracellular vesicles, cytotoxicity, Figs. S1–S3, and Tables S1 andS2.

## Data Availability

The data that support the findings of this study are available within the article and its supplementary material.
